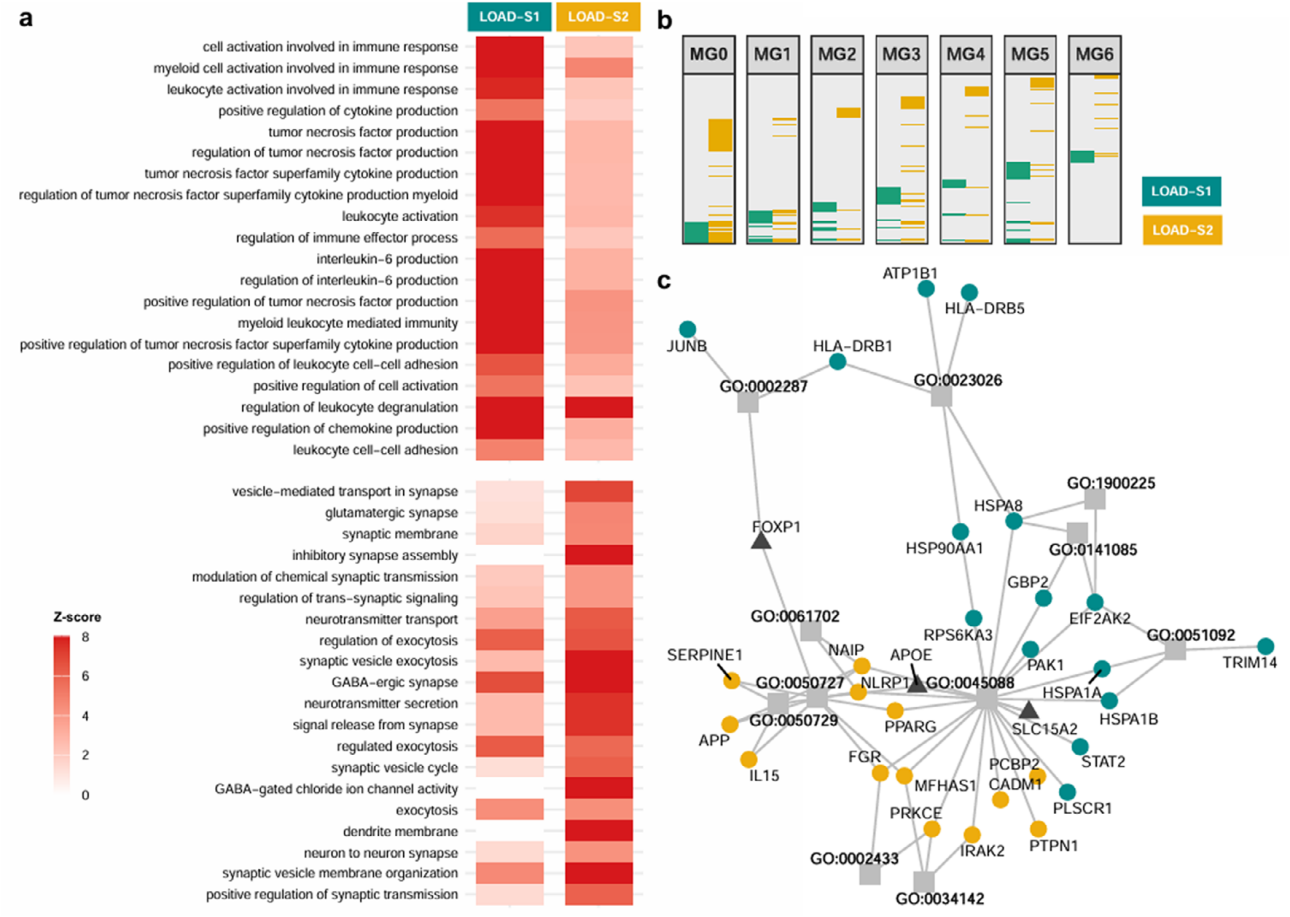# Epigenomic subtyping of late‐onset Alzheimer's disease reveals distinct microglial signatures and molecular inflammatory phenotypes

**DOI:** 10.1002/alz70855_099625

**Published:** 2025-12-23

**Authors:** Ehsan Pishva, Valentin Laroche, Rachel Cavill, Morteza P Kouhsar, Rick A Reijnders, Joshua Harvey, Adam R. Smith, Jennifer Imm, Luke Weymouth, Lachlan Ford MacBean, Giulia Pegoraro, Lars M.T. Eijssen, Jarno Koetsier, Byron Creese, Julia K. Kofler, Gunter Kenis, Daniel L.A. van den Hove, Katie Lunnon

**Affiliations:** ^1^ Maastricht University, Maastricht, Netherlands; ^2^ Maastrich University, Maastricht, Netherlands; ^3^ University of Exeter, Exeter, United Kingdom; ^4^ Brunel University of London, London, United Kingdom; ^5^ University of Pittsburgh, Pittsburgh, PA, USA; ^6^ Mental Health and Neuroscience Research Institute (MHeNs) Maastricht University, Maastricht, Limburg, Netherlands

## Abstract

**Background:**

Growing evidence suggests that the heterogeneity of late‐onset Alzheimer's disease (LOAD) plays a significant role in treatment failure. In recent years, molecular subtyping of AD has increasingly leveraged omics data to uncover distinct molecular profiles that may underlie the observed heterogeneity in disease manifestation and mechanisms. This has been particularly transformative as traditional classification methods based on clinical or pathological features alone have proven insufficient.

**Method:**

We applied data‐driven clustering to genome‐wide DNA methylation (DNAm) data from three independent post‐mortem AD brain cohorts (*n* = 831). To determine the brain cell‐type specificity of the findings identified using bulk DNAm profiles, we isolated four nuclei populations from PFC tissue of 20 donors with low neuropathology from the Brains for Dementia Research (BDR) cohort.

**Result:**

This study identified two distinct epigenetic subtypes of LOAD using genome‐wide DNAm. Data‐driven clustering revealed reproducible LOAD subtypes characterized by cell‐type‐specific DNAm profiles. Bulk transcriptomic analyses further emphasize divergent biological mechanisms driving these subtypes, with subtype 1 (LOAD‐S1) enriched in immune‐related processes and subtype 2 (LOAD‐S2) in neuronal and synaptic functions. Subtype‐specific microglial single‐cell transcriptional signatures revealed that LOAD‐S1 represents a state of chronic innate immune hyperactivation and impaired resolution, while LOAD‐S2 displays a dynamic inflammatory profile, balancing pro‐inflammatory signals with reparative and regulatory mechanisms (see Figure).

**Conclusion:**

These subtype‐specific immune signatures provide critical insights into the molecular heterogeneity of LOAD and highlight potential therapeutic targets tailored to the immune dysregulation observed in each subtype.